# Strongly Coupled Magnon–Plasmon Polaritons in Graphene-Two-Dimensional Ferromagnet Heterostructures

**DOI:** 10.1021/acs.nanolett.3c00907

**Published:** 2023-05-11

**Authors:** A. T. Costa, Mikhail I. Vasilevskiy, J. Fernández-Rossier, Nuno M. R. Peres

**Affiliations:** † International Iberian Nanotechnology Laboratory (INL), Av. Mestre José Veiga, 4715-330 Braga, Portugal; ‡ Department of Physics, Center of Physics (CF-UM-UP), University of Minho, Campus of Gualtar, 4710-057, Braga, Portugal; § Departamento de Física Aplicada, Universidad de Alicante, 03690 Sant Vicent del Raspeig, Spain

**Keywords:** magnons, plasmons, polaritons, FMR, 2D magnets

## Abstract

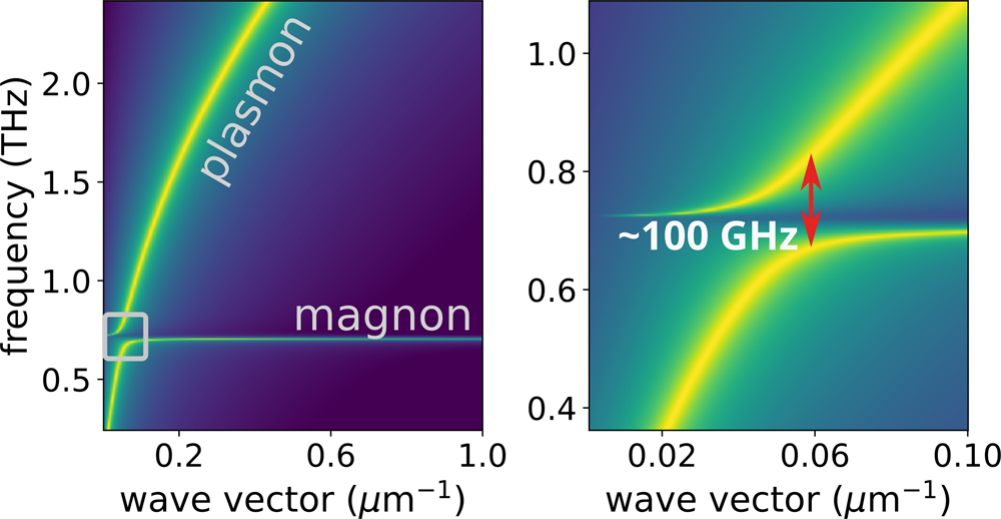

Magnons and plasmons are different collective modes, involving the spin and charge degrees of freedom, respectively. Formation of hybrid plasmon–magnon polaritons in heterostructures of plasmonic and magnetic systems faces two challenges, the small interaction of the electromagnetic field of the plasmon with the spins, and the energy mismatch, as in most systems plasmons have energies orders of magnitude larger than those of magnons. We show that graphene plasmons form polaritons with the magnons of two-dimensional ferromagnetic insulators, placed up to to half a micrometer apart, with Rabi splittings in the range of 100 GHz (dramatically larger than cavity magnonics). This is facilitated both by the small energy of graphene plasmons and the cooperative super-radiant nature of the plasmon–magnon coupling afforded by phase matching. We show that the coupling can be modulated both electrically and mechanically, and we propose a ferromagnetic resonance experiment implemented with a two-dimensional ferromagnet driven by graphene plasmons.

Magnons are the elementary excitations of every magnetically ordered system, governing their low-energy properties. Magnons have attracted renewed interest for several reasons. They can transport spin currents for applications in nondissipative spintronics,^1^ host topological order with chiral edge states,^2,3^ form exotic collective states such as Bose condensates and spin superfluids;^4^ most important for the scope of this work, they can couple to photons.^5−7^

Magnons play a particularly important role in two-dimensional (2D) magnets, as their uncontrolled thermal proliferation^8^ prevents long-range order. Thus, most prominent examples of 2D ferromagnets (2DFM), such as VI_3_,^9^ CrI_3_,^10^ and Fe_3_GeTe_2_, have a sizable gap in the magnon energy spectrum. Experimental techniques that are very successful in producing and probing magnons in bulk ferromagnets are not easily adaptable to 2D systems due to the intrinsically small sample volume. For instance, the sensitivity of the ferromagnetic resonance is limited by the ratio between sample and detector sizes. Recent proposals, such as ferromagnetic resonance force spectroscopy,^11^ address the challenge of probing submicrometer-size samples but are a long way from monolayer van der Waals magnets. Cavity magnonics^12^ has also emerged as a way of enhancing the coupling between exciting/probing fields and the magnetic sample. Rabi splittings on the order of 100 MHz have been obtained for micrometer-sized spheres on resonant microwave cavities.^13^ Further enhancement in coupling strength, leading to Rabi splittings of a few GHz, has been achieved for macroscopic-sized ferromagnets in optical^14^ and superconducting cavities.^15^

In this context, exciting and probing magnons efficiently in 2D ferromagnets remain a challenge. There are three main bottlenecks for the existing techniques. One is having a driving field of the right frequency: magnons in 2DFM have frequencies in the range ∼0.25–1 THz, whereas the highest frequencies achieved in ferromagnetic resonance (FMR) experiments are ∼700 GHz.^16^ This stems from a combination of the scarcity of microwave sources of higher frequencies and the need to match the resonance frequency of a cavity. This brings forward the second challenge, the strength of the photon–magnon coupling. The interaction of the magnetic field of light with matter is notoriously much weaker than that of the electric field.^17^ Placing the ferromagnetic sample in a resonant cavity enhances the coupling between the magnon and the cavity modes.^12^ The frequencies of those modes, however, decrease as the cavity volume increases, whereas the enhancement factor goes in the opposite direction. There is, thus, a compromise between enhancement factor and resonance frequency that limits the sensitivity of setups of this kind. This links to the third challenge, which is detector sensitivity. Again, this is limited by the smallness of light’s coupling to magnetic dipoles and puts a constraint on the minimum enhancement factor needed.

With regard to the frequency of the driving field, graphene plasmons come to mind as prime candidates. Their frequencies can be tuned essentially continuously, by gating graphene away from charge neutrality. Current experimental limits on such control set the spectral range of graphene plasmons to a few THz within the wavelength range of interest to us. Graphene plasmons have been shown to form various kinds of polaritons in van der Waals heterostructures.^18−20^ Coupling to graphene plasmons has been proposed recently as a way to probe collective excitations in superconductor surfaces,^21^ 2D superconductors,^22^ and excitons in insulators.^23^ The common theme of those works is the coupling between the strongly confined electric field associated with the graphene plasmon and the charges of the electrons in the nearby system. The coupling to spins is more subtle, since it relies on the much smaller magnetic-dipolar nature. It is known, however, that momentum and frequency matching can enhance dramatically the coupling between light and an ensemble of quantum objects.^24^ With this in mind, we have studied the coupling between graphene plasmons and 2D magnons in a van der Waals heterostructure.

Long-range ferromagnetic order in 2D is only possible in the presence of magnetic anisotropy, on account of the Mermin–Wagner theorem.^8,25^ Spin–orbit coupling breaks spin rotation symmetry, stabilizes long-range magnetic order, and opens up a gap in the magnon spectrum at zero wave vector, *q* = 0. In many cases of interest, the magnon gap in 2D ferromagnets is much larger than typical values in 3D. For instance, the magnon gap of CrI_3_ monolayers, one of the most prominent 2D magnents, has been reported to be in the 0.3–1.0 meV range.^10,26^ For some materials this value can exceed 5 meV,^27,28^ putting the lowest energy magnon in the terahertz region. On the other hand, the energy and wave vector of graphene plasmons may be tuned to match those of magnons in a 2DFM by adjusting the charge density of the graphene sheet. Thus, van der Waals heterostructures composed of 2DFM and plasmonic materials, such as graphene, may provide a platform to bridge the terahertz gap in optoelectronics. Previous attempts in this direction have been aimed at the coupling between light and the orbital magnetic moments of electrons in conducting materials,^29^ but here we focus on the spin magnetic moment, which is associated with quantum magnetism.

We consider a van der Waals heterostructure, depicted schematically in Figure 1, composed of a 2D ferromagnet with an off-plane easy axis and a graphene sheet, separated by a dielectric, such as hexagonal boron nitride, of thickness *z* and relative dielectric constant ε.

**Figure 1:**
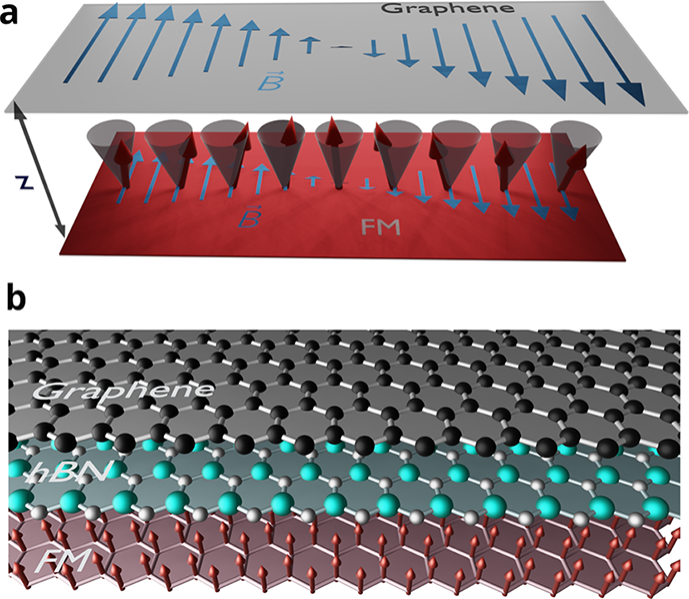
Schematic depiction of the heterostructure where strong plasmon–magnon coupling is predicted to occur. (a) Artistic rendition of the plasmon magnetic field, that emanates from the graphene layer and reaches the magnetic layer, and the precession of the spins in a magnon state, with the same wave vector as the plasmon, in the magnetic layer. (b) Scheme of the structure that would display the effect, including a graphene monolayer, a boron nitride decoupling layer, and a magnetic monolayer. The plasmon–magnon coupling is large for decoupling layers as thick as 5 μm.

The 2D ferromagnet is described with a spin Hamiltonian in the linear spin wave approximation.^30^ The in-plane magnetic field of the plasmon is coupled to the local spins of the ferromagnet via a Zeeman interaction
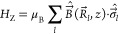
1where *R⃗*_*l*_ is the 2D vector marking the position of unit cell *l* in the 2D ferromagnet, *z* is the vertical distance between the graphene sheet and the 2D ferromagnet, and σ⃗ are the dimensionless Pauli spin matrices that relate to the spin angular momentum through .

Using the expression for the quantized field of the graphene plasmon given in ref (31), the Zeeman interaction with the transverse-magnetic (TM) plasmon magnetic field reads
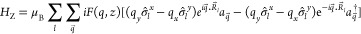
2where *a*_*q⃗*_^†^ is the creation operator for a plasmon with wave vector *q⃗* parallel to the graphene sheet. We note that the plasmon magnetic field lies in-plane, so that it generates a torque on the static magnetization. At the microscopic level, this entails the creation of magnons. The coupling strength *F*(*q*,*z*) is given by
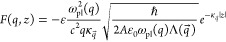
3where ℏω_pl_(*q*) is the energy of a plasmon with wave vector *q⃗*, , Λ(*q*) is the mode length of the plasmon (see the Supporting Information), and *A* is the area of the graphene sheet. The plasmon field decays exponentially, but for the range of wave vectors relevant to this work the decay length is of the order of several micrometers, thus presenting no practical concern.

We note that the coupling strength of a plasmon mode with wave vector *q* with an atomic spin *S⃗*_*l*_ is vanishingly small, as it scales with the inverse of √*A*. In contrast, for collective excitations such as magnons, it makes sense to transform the spins to a plane-wave basis
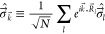
4where *k⃗* is a wave vector in the Brillouin zone of the 2DFM and *N* is the number of unit cells. After applying this transformation to eq 2 we obtain
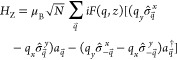
5Compared to the case of atomic spins, the magnon–plasmon coupling is enhanced by a factor √*N*, where *N* is the number of spins, resulting in a Rabi coupling that no longer depends on system size, as *N* ∝ *A*. Thus, magnon–plasmon coupling is enhanced due to the phase-matching of the plasmon field to a macroscopic number of phase-locked precessing spins.

The quantized Hamiltonian for plasmons in graphene reads
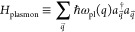
6where their energy dispersion curve is given by^32^
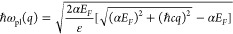
7Here, *E*_F_ is graphene’s Fermi energy, ε is the average dielectric constant of the two media surrounding the graphene sheet, α is the fine structure constant, *c* is the speed of light, and *q* is the plasmon’s (in-plane) wave vector.

To study the effect of plasmon–magnon coupling, we adopt a description of magnons in terms of linearized Holstein–Primakoff (HP) bosons^33^
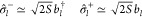
8where σ̂^+,–^ are the ladder operators acting on the spin located at site *l*; their magnitude *S* is assumed to be the same throughout the whole material. The operators *b*_*l*_^†^ and *b*_*l*_ respectively create and annihilate a localized spin flip excitation at site *l*. Assuming translation symmetry in the 2D ferromagnet, we can rewrite the HP bosons in reciprocal space. Then, the Hamiltonian for bare magnons has the form
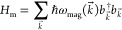
9The wave vectors *k⃗* span the Brillouin zone of the 2D ferromagnet. The function ℏω_mag_(*k⃗*) is the dispersion relation for the bare magnons. For small momenta, we have ℏω ≃ ℏω_0_ + ρ*k*^2^, where the first term is the magnon gap and the second provides the dispersion due to the exchange-driven spin stiffness ρ.

For plasmons with energies ℏω_pl_ ≈ 1 meV (thus close to that of uniform magnons in typical 2DFM) and typical graphene doping levels (*E*_F_ ≈ 100 meV), *q* ≲ 0.1 μm^–1^. This is tiny compared to the linear dimensions of the magnon Brillouin zone (∼10^4^ μm^–1^), so that the dispersion of the magnon states is negligible in that wave-vector window.

After transforming the Zeeman Hamiltonian to the HP representation in reciprocal space, it reads
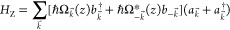
10The coupling strength is given by
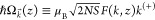
11where *z* is the distance between the graphene sheet and the 2DFM, *N* is the number of spins in the 2DFM, and *k*^(+)^ ≡ *k*_*x*_ + *ik*_*y*_. The function *F*(*k*,*z*) has been defined in eq 3. Notice that the plasmon–magnon coupling is diagonal in wave vector, meaning that each bare plasmon of wave vector *k⃗* couples only to magnons with the same wave vector. We notice in passing that the quantized treatment of both plasmons and magnons that we adopt here can be used to model intrinsically quantum phenomena, such as spontaneous emission of a plasmons due to a spin flip, or the dynamics of non-Glauber states, which are relevant for cavity quantum magnonics. These are out of reach of the classical description adopted in previous works.^18^

If the terms proportional to *b*_–*k⃗*_*a*_*k⃗*_ and *b*_*k⃗*_^†^*a*_*k⃗*_^†^ in eq 10 are neglected, the remaining Hamiltonian can be mapped onto a single-particle problem, leading to approximate analytic forms for the dispersion relations of the two hybrid plasmon-magnon modes
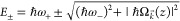
12where . This equation predicts a gap opening of magnitude ℏΩ_*k⃗*_(*z*) at the crossing frequency where the plasmon–magnon detuning ω_–_ vanishes.

In the following we treat the complete magnon–plasmon Hamiltonian, including the nonconserving terms *ba* and *a*^†^*b*^†^, by analyzing the plasmon Green function (see the Supporting Information for details), which can be probed in near-field optical experiments. Since the plasmon–magnon coupling is linear, the equations of motion for all Green functions can be solved analytically. Their explicit expressions are given in the Supporting Information. Here we will highlight the most relevant features by plotting the plasmon spectral density, , that is of course affected by the coupling to magnons.

In Figure 2a is shown the spectral density for the case where the magnon gap has ω_mag_(0) = 3 meV, corresponding to a 2DFM such as Fe_3_GeTe_2_.^34,35^ In that figure we also show the dispersion curves for the bare plasmon and magnon. The formation of a plasmon–magnon polariton with a Rabi splitting larger than 100 GHz, dramatically larger than the values reported in cavity magnonics,^12^ is apparent.

**Figure 2:**
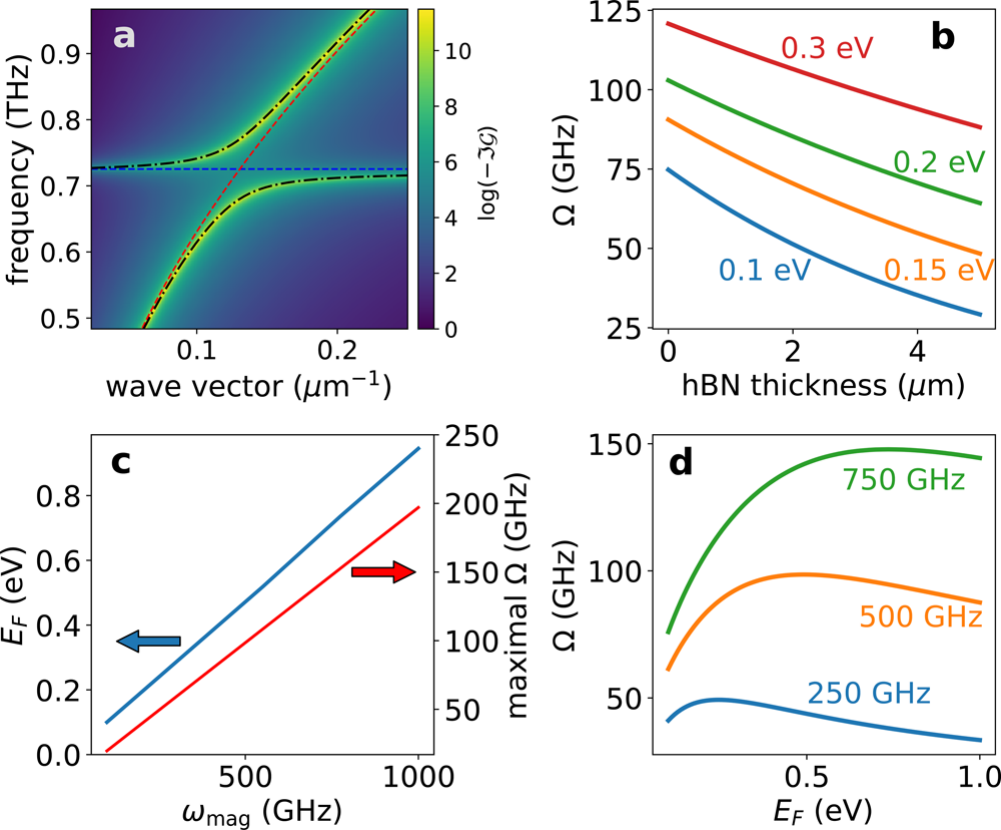
Main features of the hybrid plasmon–magnon excitation. (a) Spectral density as a function of frequency and wave vector for fixed graphene doping (*E*_F_ = 200 meV) and hBN thickness (10 nm). The magnon gap has been set at 3 meV, corresponding to a frequency of ∼0.73 THz. The dashed blue and red lines correspond to the dispersion relations of the bare magnon and plasmon, respectively. The black dot-dashed lines are the approximate dispersions of the hybrid plasmon–magnon modes given by eq 12. (b) Rabi splitting as a function of hBN thickness for different graphene doping levels (shown in the figure). The magnon gap is the same as in (a). (c) Fermi energy of graphene for which the maximum Rabi splitting is obtained, as a function of the magnon frequency (blue curve, left vertical axes), and the respective maximal splitting (red curve, right vertical axis). (d) Rabi splitting as a function of graphene gating level for different magnon frequencies (shown in the figure), at a fixed hBN thickness of 10 nm.

Interestingly, the magnitude of the Rabi coupling Ω can be tuned mechanically, by controlling the graphene–ferromagnet distance *z*, as we show in Figure 2b. In this energy range the plasmon decay rate in the direction perpendicular to the graphene layer is small, which means that the plasmon–magnon coupling is sizable even for graphene–2DFM distances on the order of 1 μm, where interlayer exchange is completely negligible.

The Rabi coupling can be further tuned electrically, controlling the graphene Fermi energy *E*_F_ with a back gate, as we show in Figure 2b,c for three different 2D ferromagnets. For a given magnon energy, there is an optimal value of *E*_F_ that maximizes the Rabi coupling strength, as we show Figure 2d. We thus see that in a wide range of experimentally relevant parameters, the intrinsic magnon–plasmon Rabi coupling due to Zeeman coupling can be the larger than 50 GHz. The estimated Rabi coupling is a lower bound, coming from the intrinsic Zeeman interaction, and additional contributions to the Rabi coupling can occur when the magnon anisotropy gap is sensitive to the plasmon electric field.

We now propose to take advantage of the magnon–plasmon coupling to carry out ferromagnetic resonance of monolayers using an attenuated total reflection setup (see Figure 3a). Exciting plasmons directly with optical beams is impossible due to the kinematic mismatch between plasmons and propagating light.^32^ By placing a prism of a high dielectric constant material on top of the hBN layer, it is possible to generate evanescent waves within the hBN that will excite the surface polaritons of the heterostructure. Whenever the in-plane component of the wave vector of light matches that of a polariton with the same frequency, there is a dip in the reflected intensity. The in-plane wave vector can be controlled via the incidence angle. With this setup, it is possible to excite polaritons whose wave vectors and frequencies lie between the light cones inside hBN and the dielectric of which the prism is made. Germanium, for instance, would be a convenient material to use for the prism. It is transparent to electromagnetic radiations of frequencies below 1 THz and its relative dielectric constant within the same frequency range is ε_Ge_ ≈ 16.^39^

**Figure 3:**
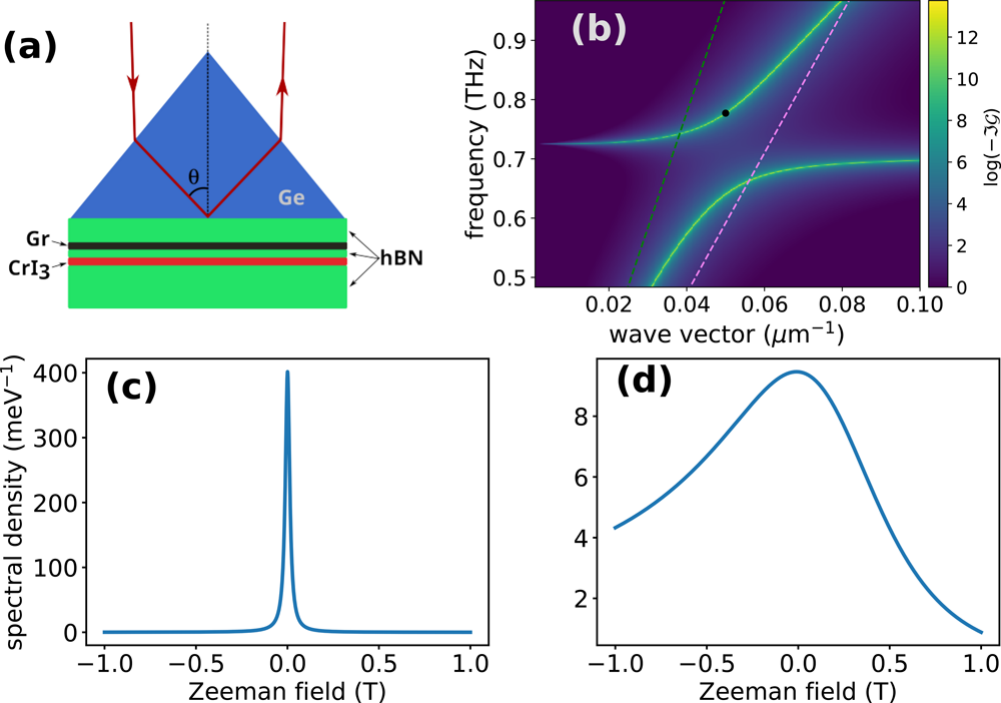
Attenuated total reflection experiment to probe the plasmon–magnon coupling. (a) Scheme of the setup. (b) Spectral density as a function of wave vector and frequency for a magnon energy of 3 meV and graphene doping corresponding to a Fermi energy of 500 meV. The spectral window probed by this experiment lies between the light dispersion relations within hBN (green dashed line) and germanium (violet dashed line). (c) Spectral density for the wave vector and frequency indicated by the black dot in (b), as a function of and external magnetic field perpendicular to the plane of the heterostructure. The plasmon lifetime has been chosen as ∼5 ns, in line with the intrinsic lifetimes given in ref (36). (d) Effect of experimentally determined plasmon (40 ps) and magnon (2 ns) lifetimes on the optically detected ferromagnetic resonance, as discussed in the main text.

In Figure 3a we plot the spectral density for a magnon gap of ℏω_0_ = 3 meV and graphene gating voltage corresponding to *E*_F_ = 500 meV. The dispersions for light inside hBN and germanium are plotted as dashed lines, to mark the spectral region probed by the experiment. The black dot in Figure 3b marks the point at which the plasmon–magnon spectral density is probed. By applying an external magnetic field perpendicular to the structure, we shift the magnon energy, thereby changing the spectral density and, consequently, the device’s reflection coefficient. In Figure 3c we plot the spectral density, for a fixed wave vector and frequency, as a function of the external magnetic field. The sharp peak heralds the magnetic nature of the polariton being probed in this setup. Finite plasmon and magnon lifetimes will broaden the resonance peak and reduce the contrast in the reflection coefficient of the device we propose. Recent experimental results^37^ indicate that graphene plasmon-polaritons can have lifetimes in the vicinity of 40 ps at a temperature of 60 K. Magnons in bulk CrI_3_, on the other hand, can have lifetimes of up to 2 ns for temperatures below the ferromagnetic transition (∼60 K).^38^ To assess the impact of finite excitation lifetime on our results, we introduced a phenomenological broadening to the bare plasmon and magnon Green functions, to mimic the experimentally estimated lifetimes. The result is shown in Figure 3d. As expected, the resonance peak is considerably broader than before, but the contrast is still large enough to allow detection of the resonance, provided the range of magnetic fields is wide enough. Thus, our proposal would permit tackling the three issues that make FMR in 2D magnets challenging and could open a new venue to explore collective spin excitations in 2D systems.

## Supporting Information Available

Descriptions of the quantization procedure for graphene plasmons and the solution of the coupled equations of motion for the plasmon and magnon Green functions and an example of plasmon spectral density showing the hybrid modes for realistic values of plasmon and magnon lifetimes (PDF)
