# Supplementary material for: Strongly Coupled Magnon–Plasmon Polaritons in Graphene-Two-Dimensional Ferromagnet Heterostructures
Source: Nano Lett. 2023 May 11;23(10):4510–5. doi: 10.1021/acs.nanolett.3c00907 (PMC10214485; doi:10.1021/acs.nanolett.3c00907)
Supplement: Supplementary file 1 — nl3c00907_si_001.pdf [file nl3c00907_si_001.pdf]

Supporting information for Strongly coupled magnon-plasmon polaritons in graphene- two-dimensional ferromagnet heterostructures

A. T. Costa,*,† Mikhail I. Vasilevskiy,†,‡ J. Fernández-Rossier,†,¶ and Nuno M. R. Peres†,‡

†*International Iberian Nanotechnology Laboratory (INL), Av. Mestre José Veiga, 4715-330 Braga, Portugal*

‡*Department of Physics, Center of Physics (CF-UM-UP), University of Minho, Campus of Gualtar, 4710-057, Braga, Portugal*

¶*On leave from Departamento de Física Aplicada, Universidad de Alicante, 03690, Sant Vicent del Raspeig, Spain*

E-mail: antonio.costa@inl.int

Quantization of the plasmon's electromagnetic field

The plasmon's magnetic field operator $\hat{\vec{B}}$ at a point $(\vec{r}, z)$ is

$$\hat{\vec{B}}(\vec{r}, z) = \sum_{\vec{q}} \left[\vec{B}_{\vec{q}}(\vec{r}, z) a^{\dagger}_{\vec{q}} + \vec{B}^{*}_{\vec{q}}(\vec{r}, z) a_{\vec{q}} \right], \tag{1}$$

where $a^{\dagger}_{\vec{q}}, a_{\vec{q}}$ are the creation and annihilation operators for the plasmon's photonic component with wave vector $\vec{q}$, and the coefficients $\vec{B}_{\vec{q}}(\vec{r}, z)$ can be derived from those appearing

in the operator for the vector potential,[1]

$$\vec{\mathrm{A}}_{\vec{q}}(\vec{r},z)=\sqrt{\frac{\hbar}{2A\epsilon_0\omega_{\mathrm{pl}}(q)\Lambda(\vec{q})}}e^{i\vec{q}\cdot\vec{r}}\times\begin{cases}\left(i\frac{\vec{q}}{q}-\frac{q}{\kappa_{1,\vec{q}}}\hat{z}\right)e^{-\kappa_{1,\vec{q}}z}, & z>0,\\ \left(i\frac{\vec{q}}{q}+\frac{q}{\kappa_{2,\vec{q}}}\hat{z}\right)e^{\kappa_{2,\vec{q}}z}, & z<0.\end{cases}\tag{2}$$

A is the area of the graphene sheet, which is assumed to occupy the plane z_0. The dispersion relation for the graphene plasmon, $\omega_{\mathrm{pl}}(q)$, can be found in the main text (eq. 7). The mode length $\Lambda(q)$ is defined as

$$\Lambda(\vec{q})=\frac{[\omega_{\mathrm{pl}}(q)c]^2}{2}\left(\frac{\epsilon_1^2}{\kappa_{1,\vec{q}}^3}+\frac{\epsilon_2^2}{\kappa_{2,\vec{q}}^3}\right)+\frac{i}{2\epsilon_0}\left[\frac{\sigma(\omega_{\mathrm{pl}}(q))}{\omega_{\mathrm{pl}}(q)}+\left.\frac{\partial\sigma}{\partial\omega}\right|_{\omega_{\mathrm{pl}}(q)}\right],\tag{3}$$

where $\sigma(\omega)$ is the conductivity of graphene, ϵ_0 is the permittivity of free space and ϵ_j are the relative permittivities of the media surrounding graphene. Also,

$$\kappa_{j,\vec{q}}\equiv\sqrt{q^2-\epsilon_j\frac{\omega_{\mathrm{pl}}^2(q)}{c^2}},\tag{4}$$

The coefficients of the expansion of the magnetic field operator into normal modes with wave vector $\vec{q}$ is

$$\vec{B}_{\vec{q}}(\vec{r},z)=\nabla\times\vec{\mathrm{A}}_{\vec{q}}(\vec{r},z)=iF(q,z)e^{i\vec{q}\cdot\vec{r}}\left(q_y\hat{x}-q_x\hat{y}\right),\tag{5}$$

with

$$F(q,z)\equiv-\epsilon_1\frac{\omega_{\mathrm{pl}}^2(q)}{c^2q\kappa_{1,\vec{q}}}\sqrt{\frac{\hbar}{2A\epsilon_0\omega_{\mathrm{pl}}(q)\Lambda(\vec{q})}}e^{-\kappa_{1,\vec{q}}z}\tag{6}$$

for $z>0$, where we assume the 2D ferromagnet to be placed.

Plasmon Green function

The two-time, retarded Green function of the plasmon can be defined as

$$\mathcal{G}(\vec{k},\vec{k}';t) \equiv \left\langle\!\!\left\langle a_{\vec{k}}(t); a^{\dagger}_{\vec{k}'} \right\rangle\!\!\right\rangle \equiv -i\theta(t) \left\langle \left[a_{\vec{k}}(t), a^{\dagger}_{\vec{k}'} \right] \right\rangle , \tag{7}$$

where time evolution is determined in the Heisenberg representation. Wherever the time argument of an operator is omitted it should be taken as $t = 0$. It is straightforward to show that, since the system is translationally invariant, $\mathcal{G}(\vec{k},\vec{k}';t) = \mathcal{G}(\vec{k};t)\delta_{\vec{k},\vec{k}'}$. The plasmon Green function obeys the equation of motion,

$$i\hbar\frac{d}{dt}\mathcal{G}(\vec{k},\vec{k}';t) = \delta(t) \left\langle \left[a_{\vec{k}}, a^{\dagger}_{\vec{k}'} \right] \right\rangle + \left\langle\!\!\left\langle \left[a_{\vec{k}}, H \right](t); a^{\dagger}_{\vec{k}'} \right\rangle\!\!\right\rangle . \tag{8}$$

But,

$$\left[a_{\vec{k}}, H_{\rm pl} \right] = \hbar\omega_{\rm pl}(k) a_{\vec{k}}, \tag{9}$$

$$\left[a_{\vec{k}}, H_{\rm Z} \right] = \Omega^{*}_{-\vec{k}}(z) b_{-\vec{k}} + \Omega_{\vec{k}}(z) b^{\dagger}_{\vec{k}}. \tag{10}$$

Thus,

$$i\hbar\frac{d}{dt}\mathcal{G}(\vec{k},\vec{k}';t) = \delta(t)\delta_{\vec{k},\vec{k}'} + \hbar\omega_{\rm pl}(k)\mathcal{G}(\vec{k},\vec{k}';t) + \Omega^{*}_{-\vec{k}}(z) \left\langle\!\!\left\langle b_{-\vec{k}}(t); a^{\dagger}_{\vec{k}'} \right\rangle\!\!\right\rangle + \Omega_{\vec{k}}(z) \left\langle\!\!\left\langle b^{\dagger}_{\vec{k}}(t); a^{\dagger}_{\vec{k}'} \right\rangle\!\!\right\rangle . \tag{11}$$

Thus, the equation of motion for the plasmon Green function is one of a (closed) system of coupled equations which also involves the magnon Green function,

$$G(\vec{k},\vec{k}';t) \equiv \left\langle\!\!\left\langle b_{\vec{k}}(t); b^{\dagger}_{\vec{k}'} \right\rangle\!\!\right\rangle \equiv -i\theta(t) \left\langle \left[b_{\vec{k}}(t), b^{\dagger}_{\vec{k}'} \right] \right\rangle , \tag{12}$$

and mixed plasmon-magnon Green functions, such as

$$\left\langle\!\!\left\langle b_{-\vec{k}}(t); a^{\dagger}_{\vec{k}'} \right\rangle\!\!\right\rangle \equiv -i\theta(t) \left\langle \left[b_{-\vec{k}}(t), a^{\dagger}_{\vec{k}'} \right] \right\rangle , \tag{13}$$

$$\left\langle\!\left\langle b^\dagger_{\vec{k}}(t); a^\dagger_{\vec{k}'} \right\rangle\!\right\rangle \equiv -i\theta(t) \left\langle \left[b^\dagger_{\vec{k}}(t), a^\dagger_{\vec{k}'} \right] \right\rangle. \tag{14}$$

After Fourier transforming to the frequency domain and some subsequent algebra, we obtain

$$\mathcal{G}(\vec{k}, \vec{k}', \mathcal{E}) = \frac{\delta_{\vec{k},\vec{k}'}}{\mathcal{E} - \hbar\omega_{\rm pl}(q) - \Sigma_{\rm pl}(\vec{k}, \mathcal{E})}, \tag{15}$$

where

$$\Sigma_{\rm Pl}(\vec{k}, \mathcal{E}) \equiv |\Omega_{\vec{k}}(z)|^2 \left(\frac{1}{\mathcal{E} - \hbar\omega_{\rm mag}(\vec{k})} - \frac{1}{\mathcal{E} + \hbar\omega_{\rm mag}(-\vec{k})} \right) \times$$
$$\left[1 + |\Omega_{\vec{k}}(z)|^2 \left(\frac{1}{\mathcal{E} - \hbar\omega_{\rm mag}(\vec{k})} - \frac{1}{\mathcal{E} + \hbar\omega_{\rm mag}(-\vec{k})} \right) \frac{1}{\mathcal{E} + \hbar\omega_{\rm pl}(k)} \right]^{-1}. \tag{16}$$

If the magnon dispersion relation is reciprocal, i.e., $\omega_{\rm mag}(\vec{k}) = \omega_{\rm mag}(-\vec{k})$,

$$\Sigma_{\rm pl}(\vec{k}, \mathcal{E}) \equiv 2\hbar\omega_{\rm mag}(k)|\Omega_{\vec{k}}(z)|^2 \left[\mathcal{E}^2 - (\hbar\omega_{\rm mag}(\vec{k}))^2 \right]^{-1} \times$$
$$\left\{ 1 + 2\omega_{\rm mag}(\vec{k})|\Omega_{\vec{k}}(z)|^2 \left[\mathcal{E}^2 - (\hbar\omega_{\rm mag}(\vec{k}))^2 \right]^{-1} [\mathcal{E} + \hbar\omega_{\rm pl}(k)]^{-1} \right\}^{-1}. \tag{17}$$

We also obtain the magnon Green function,

$$G(\vec{k}, \vec{k}'; \mathcal{E}) = \frac{\delta_{\vec{k},\vec{k}'}}{\mathcal{E} - \hbar\omega_{\rm mag}(\vec{k}) - \Sigma_{\rm mag}(\vec{k}; \mathcal{E})}, \tag{18}$$

where

$$\Sigma_{\rm mag}(\vec{k}; \mathcal{E}) \equiv 2\hbar\omega_{\rm pl}(k)|\Omega_{\vec{k}}(z)|^2 \left[\mathcal{E}^2 - (\hbar\omega_{\rm pl}(k))^2 \right]^{-1} \times$$
$$\left\{ 1 + 2\hbar\omega_{\rm pl}(k)|\Omega_{\vec{k}}(z)|^2 \left[\mathcal{E}^2 - (\hbar\omega_{\rm pl}(k))^2 \right]^{-1} \left[\mathcal{E} + \hbar\omega_{\rm mag}(-\vec{k}) \right]^{-1} \right\}^{-1}. \tag{19}$$

Effect of finite plasmon and magnon lifetimes

Bare plasmon and magnon lifetimes are modeled as purely imaginary constant self-energies added to the energies of the bare excitations. This results in broadening of the corresponding hybrid modes, as shown in Fig. 1. Recent experiments have estimated the lifetimes of graphene plasmons at a temperature of 60 K to be around 40 ps.[2] Magnons in CrI_3 have a much longer lifetime of around 2 ns, as estimated experimentally in Ref.[3]

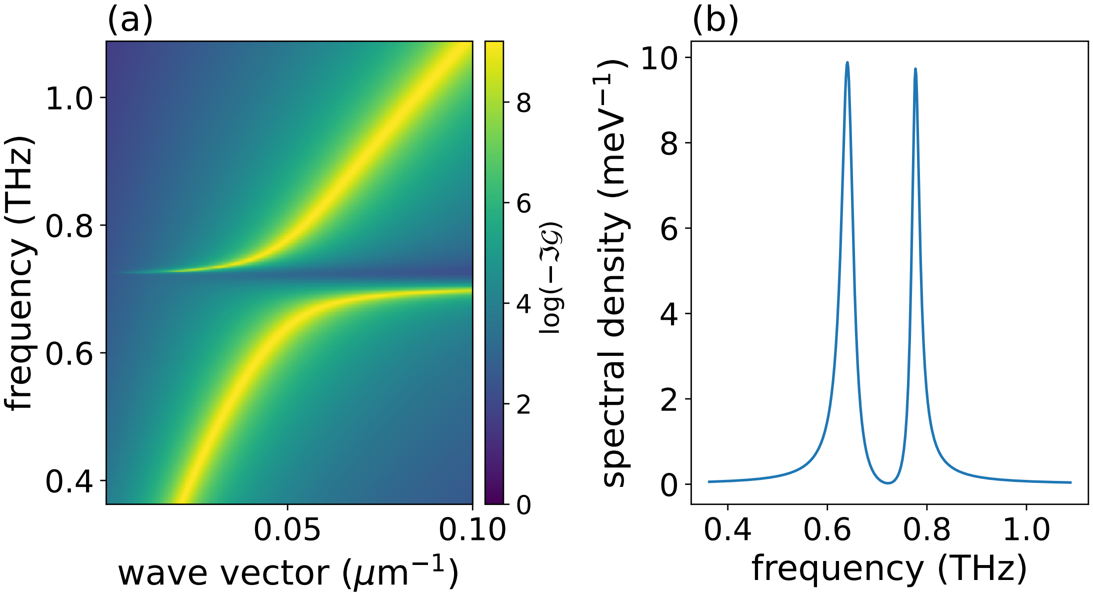

Figure 1: Plasmon spectral density as a function of wave vector and frequency for a magnon energy of 3 meV and graphene doping corresponding to a Fermi energy of 500 meV. The plasmon lifetime has been chosen as 40 ps and the magnon lifetime is 2 ns, as discussed in the text.

References

(1) Henriques, J. C. G.; Amorim, B.; Peres, N. M. R. Exciton-polariton mediated interaction between two nitrogen-vacancy color centers in diamond using two-dimensional transition metal dichalcogenides. *Phys. Rev. B* **2021**, *103*, 085407.

(2) Ni, G. X.; McLeod, A. S.; Sun, Z.; Wang, L.; Xiong, L.; Post, K. W.; Sunku, S. S.;

Jiang, B.-Y.; Hone, J.; Dean, C. R.; Fogler, M. M.; Basov, D. N. Fundamental limits to graphene plasmonics. *Nature* **2018**, *557*, 530.

(3) Jonak, M.; Walendy, E.; Arneth, J.; Abdel-Hafiez, M.; Klingeler, R. Low-energy magnon excitations and emerging anisotropic nature of short-range order in CrI_3. *Phys. Rev. B* **2022**, *106*, 214412.
